# Effects of Workplace Violence on Emergency Nurses’ Health: A Mediating and Moderating Role of Occupational Stress and the Work Environment

**DOI:** 10.1155/jonm/8813003

**Published:** 2025-12-18

**Authors:** Xiaoli Chen, Hao Zhang, Luying Zhong, Dongmei Diao, Ling Zhu, Lei Ye

**Affiliations:** ^1^ Department of Emergency Medicine, West China Hospital, Sichuan University/West China School of Nursing, Sichuan University, Chengdu, China, scu.edu.cn; ^2^ Disaster Medical Center, Sichuan University, Chengdu, China, scu.edu.cn; ^3^ Nursing Department of West China Hospital, Sichuan University, Chengdu, China, scu.edu.cn

**Keywords:** emergency nurse, health, occupational stress, somatic symptoms, workplace violence

## Abstract

**Background:**

Emergency nurses are at the highest risk of experiencing workplace violence, which can negatively impact their physical and psychological health. However, the mechanisms by which workplace violence affects nurses’ health remain unclear. Therefore, further exploration of potential moderating factors is required to understand the impact of workplace violence on nurses’ health.

**Objectives:**

This study applied the effort–reward imbalance (ERI) model to investigate the mediating role of occupational stress in the relationship between workplace violence and health among emergency nurses, with the work environment serving as a moderating factor.

**Design:**

A cross‐sectional study was conducted.

**Setting and participants:**

A questionnaire survey was administered to 1,540 emergency nurses from 30 tertiary hospitals in China between December 26, 2023, and January 18, 2024.

**Methods:**

An online questionnaire was used to obtain demographic information, along with data concerning workplace violence, work environment, occupational stress, and somatic symptoms. Latent moderate structural equations were employed to analyze the moderated mediation model, and the bootstrap method was used to test for mediating effects.

**Results:**

The path coefficient from workplace violence to occupational stress was significant (*β* = 0.526, *P* < 0.01), as was the coefficient from occupational stress to somatic symptom (*β* = 0.655, *P* < 0.01). Additionally, the direct effect of workplace violence on somatic symptoms was significant (*β* = 0.090, *P* < 0.01), with the mediating effect of occupational stress estimated at 0.433 (95% confidence interval [CI]: 0.374–0.493). The mediating effect of occupational stress on the relationship between workplace violence and somatic symptoms accounted for 79.2% of the total effect, indicating a significant mediation. Finally, the work environment moderated the impact of workplace violence on occupational stress (*β* = 0.063, *P* < 0.01).

**Conclusions:**

A moderated mediation model was developed, with occupational stress mediating the impact of workplace violence on the health of emergency nurses. The findings suggest the need for other measures to reduce the impact of workplace violence on emergency nurses’ health, as well as interventions to reduce occupational stress. The combined effects of the work environment and occupational stress emphasize the need for comprehensive interventions to create a supportive workplace for nurses, thereby reducing the fit between emergency nurses and a poor working environment.

## 1. Introduction

According to the World Health Organization (WHO), workplace violence (WPV) refers to instances in which employees in their work environment are subjected to nonphysical (e.g., verbal abuse and threats) and physical (e.g., physical attacks) violence that pose explicit or implicit challenges to their safety and well‐being [1]. Globally, between 19.3% and 36.4% of hospital staff experience WPV [2, 3]. Despite efforts by various authorities to reduce WPV in healthcare settings, its incidence remains high and continues to increase [4–6]. Notably, the emergency department (ED) is a clinical area associated with the highest WPV risk [7, 8]. Based on reviewed data, 55.7% of emergency nurses reported exposure to some form of WPV [7]. Emergency nurses face the highest risk of experiencing WPV compared with nurses in other healthcare settings (any violence, odds ratio [OR] = 34.63; nonphysical violence, OR = 32.62) [9]. These data indicate that emergency nurses face the highest risk of WPV among healthcare workers. For emergency nurses, care work encompasses physical and mental exertions, as well as psychological and emotional inputs. However, exposure to WPV significantly intensifies this effort, placing nurses in a state of chronic high stress.

The negative impact of WPV on nurses’ health, including physical and psychological symptoms, has been well documented [10]. Healthcare workers exposed to WPV are prone to coronary heart disease and cardiovascular disorders [11]. Furthermore, WPV can lead to short‐ and long‐term emotional and psychological effects. Experiences of WPV contribute to stress, sleep disorders, anxiety, and depression among healthcare workers [12–14]. Notably, the psychological impact of WPV does not diminish or resolve over time, at least in the short term. A study found that symptoms of depression and anxiety among medical staff in China who experienced WPV did not decrease over time and peaked 1 month after exposure [15]. Some nurses experience moderate‐to‐severe psychological reactions for 6 months or even more than a year following exposure to WPV [16]. The symptoms they experience are primary indicators of their health problems, particularly anxiety and depression. According to the Diagnostic and Statistical Manual of Mental Disorders (DSM‐5), anxiety and depression are the two most common mental disorders. The DSM‐5 states that somatic symptoms comorbid with anxiety and depression can exacerbate the severity and complexity of somatic components [17]. Therefore, understanding the mechanisms through which WPV affects emergency nurses’ health and identifying potential intervention targets are imperative.

Occupational stress refers to the physiological and psychological reactions that arise when the demands of an individual’s job do not match their ability, resources, or needs. This stems from the effort–reward imbalance (ERI) model, characterized by high effort and low rewards. Studies have shown that occupational stress is a significant risk factor for work‐related illnesses, particularly coronary heart disease, diabetes, anxiety, depression, and physical pain [18–21]. Occupational stress‐related health damage among medical staff has become a focus of current occupational health studies. WPV is a risk factor for occupational stress among emergency nurses [22]. Studies have shown that occupational stress mediates the relationship between perceived stress and psychological disorders among nurses [23]. Although WPV is a primary work stressor for emergency nurses, whether occupational stress moderates the impact of WPV on their health remains unclear.

The actual physiological and psychological impacts of occupational stress vary according to individual characteristics and the work environment. Specifically, the nursing work environment refers to the conditions that provide nurses with autonomy, responsibility, and job satisfaction while delivering patient care services [24]. Reportedly, a positive nursing work environment effectively improves nurses’ health outcomes, enthusiasm, and work engagement [25–27]. Studies have also demonstrated that the work environment significantly influences nurse burnout, turnover intention, and quality of nursing care [28, 29]. WPV poses a direct threat to the respect and recognition nurses deserve for their efforts, while an unsafe environment severely undermines occupational safety, generating profound anxiety and uncertainty about their future [30, 31]. However, the impact of the work environment on nurses’ health has been overlooked. Considering the variations in organizational perceptions and work experiences among nurses in various clinical departments, exploring the work environment’s influence on emergency nurses, in combination with the nursing work environment in the ED, is necessary.

Previous research has shown that occupational stress may mediate the relationship between perceived stress and mental health, as it represents a mechanism through which work stressors negatively impact health by interfering with occupational stress. However, the mediating role of occupational stress between WPV and nurse health remains unclear. The work environment has also been identified as a potential moderating factor in the relationship between work stressors and nurse health, with a good nursing work environment effectively improving nurse outcomes. While studies have confirmed that the work environment can moderate the impact of WPV on emotional exhaustion, no research has explored how it moderates WPV’s effect on occupational stress among emergency nurses.

The ERI model was developed based on an analysis of the correlation between work‐related psychosocial stress and health [32]. In particular, the ED is a high‐incidence setting for WPV and simultaneously represents a typical “high‐demand, low‐control” environment. Nurses in this context expend considerable physical and psychological efforts but often fail to receive the respect and recognition they deserve owing to heavy workloads and misunderstandings from patients and their families. Within the ERI model, “effort” encompasses physical and mental exertions, as well as psychological and emotional investments. Exposure to WPV imposes a substantial, involuntary, and negative emotional and psychological burdens on emergency nurses, significantly intensifying the “effort” dimension and subjecting them to prolonged high levels of occupational stress [33, 34]. WPV represents a severe breach of this social contract, creating a profound imbalance between nurses’ contributions and their rewards. The ERI model transcends simplistic attributions of stress solely to workload, recognizing that WPV fundamentally alters the nature of the nurses’ work environment. These structural characteristics align closely with the scenarios depicted in the ERI model, endowing the model with high ecological validity in the context of the ED. Many studies have supported the current work stress theory hypothesis regarding the correlation between ERI at work and cardiovascular disease risk, mental disorders, neurosis, and alcohol dependence [35–38].

Based on the ERI model, this study focuses on the mediating role of occupational stress in the relationship between WPV and emergency nurses’ health. Previous research has established relationships between WPV, occupational stress, and nurses’ health. However, the mediating role of occupational stress in the relationship between WPV and nurses’ health remains unclear. Therefore, this study explored the mediating effect of occupational stress on the relationship between WPV and health among emergency nurses using the work EFI model and considering occupational factors and the work environment. We hypothesized that WPV and occupational stress can directly predict emergency nurses’ health, occupational stress mediates the relationship between WPV and health, and the work environment moderates WPV’s impact on occupational stress and health (Appendix S1).

## 2. Methods

### 2.1. Study Design and Participants

A cross‐sectional survey was conducted with 1,551 emergency nurses selected from 30 tertiary hospitals in China from December 26, 2023, to January 18, 2024. Participants were recruited using stratified cluster sampling, with stratification based on the geographical regions of China (Northeast, North, Central, South, Southwest, Northwest, and East China). Within each region, 2–3 provincial capital cities per layer and 2–3 tertiary hospitals per capital city were selected. The inclusion criteria required participants to be registered nurses with at least 1 year of work experience in the ED, ensuring adequate exposure to and full understanding of WPV. Nurses with a history of mental illness, as well as those on maternity leave or breastfeeding during the study period, were excluded. Based on the results of a preliminary survey, the required sample size for this study was calculated to be 1,330 cases using a power analysis, with a significance level of 0.05 and a power of 0.8. The study design and reporting were conducted according to the Strengthening the Reporting of Observational Studies in Epidemiology guidelines [39].

### 2.2. Measures

A demographic survey of emergency nurses was conducted using a basic information questionnaire. The questionnaire collected data on demographic characteristics (gender, age, education level, and marital status), lifestyle habits (smoking, alcohol consumption, and sleep status), and occupational characteristics (work experience, weekly work hours, frequency of night shifts, and monthly income status).

The survey employed a WPV questionnaire [40] adapted from the 2005 National Survey of the Work and Health of Nurses (Statistics [41]) and a previous study [42]. Specifically, the questionnaire was administered in Chinese and assessed the frequency of WPV experienced by emergency nurses over the past 1 year. It comprised the following four items with two subscales: (a) nonphysical violence from patients and/or families, (b) nonphysical violence from employers and/or colleagues, (c) physical violence from patients and/or families, and (d) physical violence from employers and/or colleagues. Responses were rated on a 7‐point Likert scale (0 = never; 6 = every day), with higher average scores indicating more frequent WPV experience. Cronbach’s *α* coefficient of the questionnaire was calculated to be 0.71.

The Chinese version of the Nursing Work Environment Scale [43], developed by Shao Jing et al. based on the Nursing Work Index—Revised [44] and the Practice Environment Scale [45], was used to assess the work environment. It includes 26 items in seven dimensions as follows: career development, leadership and management, nurse–physician relationships, recognition atmosphere, professional autonomy, basic guarantees, and adequate staffing. Responses were rated on a 6‐point Likert scale (1 = *strongly disagree*; 6 = *strongly agree*), with total scores ranging from 26 to 156. Higher scores indicate greater satisfaction with the work environment. Overall Cronbach’s *α* coefficient for the scale was 0.946, while Cronbach’s *α* coefficients for the seven dimensions ranged from 0.799 to 0.896. In the present study, Cronbach’s *α* coefficient of the scale was 0.981.

The Occupational Stress Scale [46], translated into Chinese [47] and guided by the ERI model, was used to evaluate occupational stress among emergency nurses. It was administered in Chinese and comprised 22 items, including 6 effort, 11 reward, and 5 overcommitment items. Scores ranged from 1 to 5 (1 = *strongly disagree*; 5 = *strongly agree*), with higher scores indicating greater levels of occupational stress. Cronbach’s *α* coefficients of the three dimensions of the scale were 0.75, 0.89, and 0.84, while Cronbach’s *α* coefficient of the questionnaire was 0.82. In this study, Cronbach’s *α* coefficient of the scale was 0.957.

The self‐reported Somatic Symptom Scale China [48] was administered in Chinese and used to measure the severity of somatic symptom disorders (physical [10 items] and psychological [10 items] symptoms) in emergency nurses over the past 6 months. It includes 20 items in the following four dimensions: physical symptoms, anxiety (assessing symptoms of generalized anxiety), depression (evaluating depressive mood and anhedonia), and anxiety and depressive disorders (identifying the coexistence of both anxiety and depressive disorders). Participants rated the occurrence of each symptom on a 4‐point scale (1 = *not present*; 2 = *present for several days or tolerable*; 3 = *present half of the time*, with hope for relief; and 4 = *almost every day or difficult to tolerate*). Total scores determined the severity of somatic symptom disorder, with scores of 20–29, 30–39, 40–59, and ≥ 60 indicating normal, mild, moderate, and severe symptoms, respectively. The correlation coefficients of the dimensions and total scale ranged from 0.76 to 0.88, with a test–retest reliability of 0.9. Furthermore, the diagnostic cutoff value was 36, with a sensitivity and specificity of 0.97 and 0.96, respectively. In the present study, Cronbach’s *α* coefficient of the scale was 0.974.

### 2.3. Data Collection

An electronic version of the survey questionnaire was created using Wenjuanxing—an online survey platform—and included the informed consent form and survey link. With support from the Emergency Nursing Committee of the Chinese Nursing Association, emergency nurse managers of each hospital were contacted directly to explain the study’s purpose, significance, and precautions. Nurse managers distributed the questionnaire QR code link via WeChat—an instant messaging service application—to eligible emergency nurses, along with an explanation of the research purpose and assurances regarding voluntary participation and anonymous submission. To prevent duplicate submissions, responses were restricted to one per IP address. After collecting and sorting the questionnaires, 11 responses with identical answers for all items were excluded.

### 2.4. Statistical Analysis

All statistical analyses were performed using IBM SPSS Statistics for Windows, Version 26.0 (IBM Corp., Armonk, N.Y., USA). Descriptive statistics were used to describe continuous and categorical data as mean (standard deviation [SD]) and n (%), respectively. Pearson correlation was used to explore preliminary relationships between the dimensions and factors in the model. Mplus Version 8.3 was used to conduct structural equation modeling (SEM). A simple mediation model in the Mplus software was applied for mediation analysis. The bootstrap method was used to evaluate mediating effects [49]. With the bias‐corrected bootstrap sample set to 5,000, a mediating effect was considered statistically significant if its 95% confidence interval (CI) did not include zero. A significance level of *α* = 0.05 (two‐tailed) was used. A latent moderated structural equation was used to analyze the moderated mediation model [50]. First, the root mean square error of approximation (RMSEA) of the baseline model without the interaction term was < 0.08, the values of the Tucker–Lewis index (TLI) and comparative fit index (CFI) were greater than 0.90, and the value of standardized root mean square residual (SRMR) was < 0.08. Second, the Akaike information criterion (AIC) and H0 values were comprehensively evaluated using the benchmark SEM model without latent adjustment (interaction) and the regulated intermediary SEM model with latent adjustment (interaction). Based on the H0 value, the log‐likelihood ratio test was conducted to calculate the 2LL value, that is, the difference between the likelihood ratios of the baseline and moderated mediation models. The chi‐square test for the 2LL value was significant, indicating that the moderated mediation model was appropriate [51, 52]. In addition, we use confirmatory factor analysis (CFA) to validate the structural validity of WPV, occupational stress, work environment, and somatic symptom (Appendix S2).

### 2.5. Ethical Considerations

This study was approved by the Ethics Review Committee of West China Hospital of Sichuan University (approval number: 2024.309), and participation was voluntary. All participants provided informed consent prior to participation. Participants could terminate the survey at any point if they felt uncomfortable with the options.

## 3. Results

### 3.1. Participant Demographics

A total of 1,540 emergency nurses (78.6% women and 21.4% men), with a mean age of 32.23 ± 6.80 years, from 30 hospitals were surveyed. These 30 hospitals were geographically distributed as follows: 4, 4, 4, 5, 4, 4, and 5 hospitals in Northeast, North, Central, South, Southwest, Northwest, and East China, respectively. Among the 1,540 emergency nurses, 1,309 (85%) reported experiencing physical or nonphysical violence in the past year, including 943 (61.23%) incidents of physical violence. More than half (58.3%) of the emergency nurses had received classroom‐based professional education on WPV. Table 1 presents the descriptive analyses of the demographic information, lifestyle habits, and occupational characteristics of the participants.

**Table 1:** The demographic information of participants (*n* = 1540).

Variable	Category	N (%)
Age	32.23 ± 6.79 (years old)	
Work experience	9.74 ± 7.23 (years)	
Gender	Female	1211 (78.6)
	Male	329 (21.4)
Marital status	Single	560 (36.4)
	Married	980 (63.6)
Diploma	Junior college	186 (12.1)
	Bachelor’s or above	1354 (87.9)
Professional title	Junior	907 (58.9)
	Intermediate	574 (37.3)
	Senior	59 (3.8)
Training of violence	No	642 (41.7)
	Yes	898 (58.3)
Smoking	No	1453 (94.4)
	Yes	87 (5.6)
Alcohol consumption	No	1355 (88.0)
	Yes	185 (12.0)
Dyssomnia	No	627 (40.7)
	Yes	913 (59.3)
Night shift	0 times/month	181 (11.8)
	1–4 times/month	239 (15.5)
	5–8 times/month	659 (42.8)
	> 8 times/month	461 (29.9)
Work experience	≤ 2 years	242 (15.7)
	3–10 years	742 (48.2)
	11–20 years	421 (27.3)
	≥ 21 years	135 (8.8)
Work hours	≤ 40 h	577 (37.5)
	41–48 h	780 (50.6)
	49–58 h	123 (8.0)
	≥ 59 h	60 (3.9)
Income	< 4,000 yuan/month	57 (3.7)
	4,000–5,999 yuan/mont	149 (9.7)
	6,000–7,999 yuan/mont	250 (16.2)
	8,000–9,999 yuan/mont	368 (23.9)
	≥ 10,000 yuan/mont	716 (46.5)

### 3.2. Correlation Analysis of Dimensions

The average scores for WPV, somatic symptoms, work environment, and occupational stress were 2.2 ± 1.97, 39.58 ± 13.61, 117.55 ± 24.6, and 55.55 ± 16.78, respectively (Table 2).

**Table 2:** Scores for each dimension.

Item	Minimum	Maximum	Score ($\overset{¯}{x}\pm s$)
**Workplace violence**	0	12	2.2 ± 1.97
Physical violence	0	6	0.76 ± 0.98
Emotional violence	0	6	1.44 ± 1.17
**Occupational stress**	22	110	55.55 ± 16.78
Effort	6	30	19.22 ± 5.35
Reward	11	55	23.72 ± 9.1
Over‐commitment	5	25	12.6 ± 4.74
**Nursing work environment**	26	156	117.55 ± 24.6
Career development	5	30	23.1 ± 5.26
Leadership and management	4	24	17.9 ± 4.4
Nurse‐physician relationships	4	24	18.19 ± 4.1
Recognition atmosphere	3	18	14.02 ± 2.89
Professional autonomy	4	24	18.97 ± 3.74
Basic guarantees	3	18	12.75 ± 3.68
Adequate staffing	3	18	12.62 ± 3.68
**Somatic symptom**	20	80	39.58 ± 13.61
Physical symptoms	10	40	19.13 ± 6.87
Psychological symptoms	10	40	20.46 ± 7.08

Bivariate correlation analysis showed that the correlation coefficient between WPV and occupational stress was 0.577 (*P* < 0.01). The correlation coefficients of WPV with occupational stress and somatic symptoms were 0.468 and 0.640, respectively (both *P* < 0.01). WPV, occupational stress, and somatic symptoms were significantly and positively correlated. The correlation coefficients of WPV with occupational stress, somatic symptoms, and work environment were −0.347, −0.505, and −0.370, respectively, indicating significant negative correlations (*P* < 0.01). Table 3 presents the correlations among the dimensions.

**Table 3:** Correlations among the dimensions.

	Workplace violence	Occupational stress	Work environment	Somatic symptom
Workplace violence	1	0.577^∗∗^	−0.347^∗∗^	0.468^∗∗^
Occupational stress		1	−0.505^∗∗^	0.640^∗∗^
Work environment			1	−0.370^∗∗^
Somatic symptom				1

^∗∗^ *p* < 0.01, the correlation was significant.

### 3.3. Mediation Model of the Impact of WPV on Somatic Symptoms

**Figure 1:**
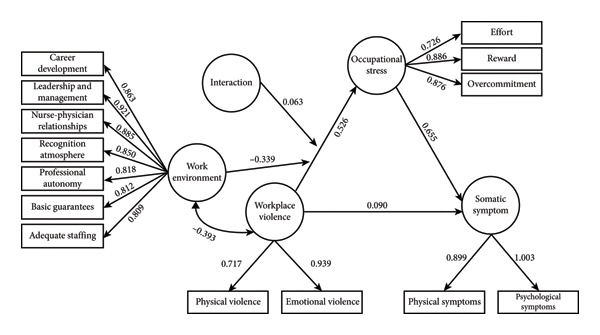
The Mediating and Moderating model of WPV on emergency nurses’ health.

We constructed mediation and moderation models in which occupational stress mediated the impact of WPV on somatic symptoms (Figure 1). The SEM demonstrated good fit: χ^2^/df = 681.321/65, RMSEA = 0.078, SRMR = 0.044, CFI = 0.969, and TLI = 0.957. Moreover, the degree of model fit was evaluated. The AIC of the latent moderated mediation decreased by 10.193 compared with the baseline model (Table 4). Furthermore, the chi‐square test of the 2LL value was significant. These findings indicated that the theoretical model used in this study demonstrated good fit and was acceptable.

**Table 4:** Fitting indicators of mediation model of the impact of workplace violence on health.

	**χ** ^2^/*df*	RMSEA	CFI	TLI	SRMR	AIC	H0	Parameters
Baseline model	681.321/65	0.078	0.969	0.957	0.044	41,690.519	−20791.259	54
Latent moderate model	/	/	/	/	/	41,680.326	−20785.163	55
Model comparison	/	/	/	/	/	10.193	0.000	/

Abbreviations: RMSEA, Root Mean Square Error of Approximation; GFI, Chi‐square Goodness of Fit Index; TLI, Tucker‐Lewis Index; SRMR, Standardized Root Mean Square Residual.

### 3.4. Mediating Effect of Occupational Stress on the Relationship Between WPV and Somatic Symptoms

As presented in Table 5, the results showed significant path coefficients from WPV to occupational stress (*β* = 0.526; *P* < 0.01), from occupational stress to somatic symptoms (*β* = 0.655; *P* < 0.01), and from WPV to somatic symptoms (*β* = 0.090; *P* < 0.01). The mediating effect of occupational stress was 0.433 (95% CI: 0.374–0.493). Additionally, the mediating effect analysis revealed that occupational stress accounted for 79.2% of the total effect between WPV and somatic symptoms. These findings indicate that WPV not only directly predicts somatic symptoms but also predicts somatic symptoms through the mediating effect of occupational stress.

**Table 5:** Mediating effect of occupational stress on the relationship between workplace violence and somatic symptoms.

Pathway	*β′*	*β*	SE	*t*	95% *CI*	
					LLCI	ULCI
Workplace Violence⟶Occupational stress	0.531	0.526	0.023	23.297^∗∗^	0.481	0.570
Occupational Stress⟶Somatic symptom	0.816	0.655	0.026	25.056^∗∗^	0.604	0.706
Workplace Violence⟶Somatic symptom	0.114	0.090	0.029	3.066^∗∗^	0.033	0.148

Abbreviations: *β′*, Non‐standardized Regression Coefficients; *β*, Standardized Regression Coefficients; SE, Standard Error.

^∗∗^ *P* < 0.01.

### 3.5. Moderating Effect of Work Environment on the Relationship Between WPV and Occupational Stress

**Figure 2:**
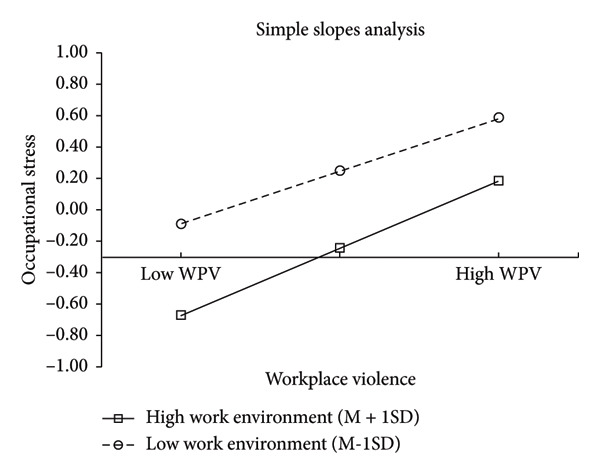
The impact of workplace violence on occupational stress moderated by different levels of working environment.

As presented in Table 6, the interaction between WPV and work environment had a significant positive predictive effect on occupational stress (*β* = 0.063; *P* < 0.01), indicating that the work environment moderates the direct impact of WPV on occupational stress. To further analyze this interaction, the work environment was categorized into high‐ and low‐level groups based on mean ± SD, and a simple slope test was conducted. The results confirmed that the impact of WPV on occupational stress was moderated by the level of the work environment. WPV had an indirect effect on somatic symptoms through occupational stress in high‐level work environments (*β* = 0.485, 95% CI: 0.413–0.557). This indirect effect was lower in low‐level work environments (*β* = 0.382, 95% CI: 0.322–0.442) (Table 7; Figure 2). WPV was found to be more likely to affect symptoms through occupational stress in a high‐level work environment than in a low‐level work environment.

**Table 6:** The moderating effect of work environment on the relationship between workplace violence and occupational stress.

	*β′*	*β*	SE	*t*	95% *CI*	
					LLCI	ULCI
Workplace violence	0.531	0.526	0.023	23.297^∗∗^	0.481	0.570
Work environment	−0.245	−0.339	0.022	−15.053^∗∗^	−0.383	−0.295
Workplace violence × work environment	0.063	0.063	0.018	3.515^∗∗^	0.028	0.098

Abbreviations: *β′*, Non‐standardized Regression Coefficients; *β*, Standardized Regression Coefficients; SE, Standard Error.

^∗∗^ *P* < 0.01.

**Table 7:** The mediating effect of occupational stress at different level of work environment.

	*β*	SE	*t*	95% *CI*	
				LLCI	ULCI
Mediating effect	0.433	0.030	14.292^∗∗^	0.374	0.493
High level work environment slope	0.594	0.039	15.418^∗∗^	0.519	0.670
Low level work environment slope	0.468	0.032	14.711^∗∗^	0.406	0.530
High level work environment indirect effect	0.485	0.037	13.192^∗∗^	0.413	0.557
Low level work environment indirect effect	0.382	0.031	12.504^∗∗^	0.322	0.442
Difference of indirect effect	0.103	0.030	3.462^∗∗^	0.045	0.162

Abbreviations: *β*, Standardized Regression Coefficients; SE, Standard Error.

^∗∗^ *P* < 0.01.

## 4. Discussion

This study examined the relationships among WPV, work environment, and occupational stress and explored the effect of WPV on emergency nurses’ health through occupational stress. The findings revealed that occupational stress serves as a significant mediator in the relationship between WPV and emergency nurses’ health, while work environment conditions moderate both the direct and indirect effects. This result contrasts with prior literature, which primarily established a direct association between WPV and adverse health outcomes but did not clarify the underlying psychological and contextual mechanisms. The divergence may be attributed to the integrated analytical approach adopted in this study, which simultaneously examined mediation and moderation effects. By identifying modifiable factors such as occupational stress and work environment conditions, this study provides a more nuanced understanding of the causal pathways and offers valuable insights for developing targeted organizational interventions.

In this study, WPV was positively correlated with somatic symptoms and affected emergency nurses’ health through direct (WPV⟶somatic symptom) and indirect (occupational stress⟶somatic symptom) pathways. This finding aligns with those of previous studies [53, 53]. Mechanism studies confirming the indirect effects of WPV on the health of nurses are scarce. The present study included occupational stress as an intermediary factor based on the ERI model at work and revealed the pathway through which occupational stress mediates the impact of WPV on nurses’ health. Occupational stress as a mediator significantly predicted nurses’ somatic symptoms, affecting both physical and psychological symptoms. This indicates that occupational stress had a significant effect on predicting nurses’ health outcomes. The effect of WPV on health through occupational stress supports the mechanism underlying the impact of life‐ and work‐related stressors on emergency nurses’ health. Occupational stress can cause physiological changes, potentially resulting in musculoskeletal disorders, such as muscle soreness and joint strain, as well as cardiovascular diseases, including hypertension and lipid abnormalities [54]. Excessive occupational stress causes inattention and memory loss among medical staff, which can lead to anxiety and depression in severe cases. Healthcare workers face stress from various aspects—including clinical and research domains—due to job‐related pressures. Negative emotions may arise when work‐related inputs—including time and energy—far exceed the rewards gained, potentially leading to depressive symptoms. Therefore, such symptoms among emergency nurses may be alleviated by reducing effort and overcommitment while increasing rewards.

Another important finding of this study is the moderating effect of the work environment on the relationship between WPV and occupational stress. The results demonstrated that WPV’s impact on occupational stress increased in healthier high‐level work environments. Higher WPV increased occupational stress more significantly in healthy work environments than in unhealthy work environments. According to the people–environment fit theory [55], fit exists between the individual and organization when at least one person and the organization can provide the resources needed by the other party, and/or the person and the organization have similar basic characteristics. The fit between people and the environment generally has a significant impact on individuals. Work experience creates a fit between emergency nurses and their work environment. When emergency nurses are frequently exposed to WPV, they tend to normalize the violence, and WPV becomes part of their work environment. However, a healthy work environment has low or no WPV. Therefore, WPV in healthy work environments creates sudden stress and has a more significant impact on occupational stress.

Work environment conditions moderated the direct and indirect effects of WPV on nurses’ health outcomes. WPV is more likely to affect symptoms through occupational stress in high‐level work environments than in low‐level work environments. This finding aligns with those of Havaei (2020), who revealed that exposure to violence in a healthy work environment was associated with higher levels of physical and mental health problems among nurses. This may be related to the mechanisms of occupational stress and people–environment fit described. Extensive literature indicates that healthy work environments are associated with good health among nurses [56–58]. The present correlation analysis demonstrated that occupational stress was negatively correlated with the work environment, indicating lower levels of occupational stress in healthier work environments. A nursing work environment refers to one in which nurses have more autonomy and responsibility in providing nursing care, including involvement in hospital affairs, nursing management capabilities and leadership styles, adequate staffing and resources, and interprofessional collaboration [24, 45]. A positive work environment enhances nursing professionalism, encourages nurses to exercise autonomy in clinical work, and improves nurses’ job satisfaction and nursing quality while reducing burnout and negative emotions such as psychological anxiety [29, 59, 60]. From an organizational perspective, nursing managers should implement relevant measures to improve the nursing work environment, reduce WPV, and prevent the normalization of WPV.

Based on the complex effects of WPV, work environment, and occupational stress on emergency nurses’ health, several key points should be considered when designing measures to manage their health. First, comprehensive and effective management measures for WPV should be implemented. Such measures require collaborations among multiple departments, including local governments, health committees, and medical institutions, to construct a comprehensive management structure system for WPV. Second, nursing managers should focus on the psychological status of nurses following WPV incidents and provide timely, effective interventions to reduce the adverse effects of WPV. Finally, nursing managers can reduce occupational stress‐related health problems among nurses by optimizing the work environment, increasing nursing labor force, and improving remuneration.

### 4.1. Limitations

This study had some limitations. First, it employed a cross‐sectional survey design, relying on a single‐time‐point survey, and did not include longitudinal data. Second, although stratified cluster sampling was used to improve the structural representation of the sample, the sampling framework was limited to specific medical institutions, which may have restricted diversity in the occupational environment and cultural backgrounds, potentially affecting the external generalization of the results. Finally, the study exclusively focused on emergency nurses. The working environment and stress characteristics of this group are highly specialized, considering the differences in WPV and work environments in different clinical departments; therefore, the results should be verified in other nurse populations. Future research could expand the sample coverage through multicenter random sampling and include nurses from different regions, hospital levels, and other departments for comparative analysis to improve generalizability. Further longitudinal studies are needed to explore the long‐term impact of WPV on nurses’ health and the regulatory mechanism of protective factors.

## 5. Conclusion

WPV is prevalent among emergency nurses, and exposure to WPV can lead to acute and long‐term physiological and psychological consequences. This study revealed the pathway through which occupational stress mediated WPV’s impact on emergency nurses’ health. WPV was found to be a stressor that triggered occupational stress among emergency nurses. Healthier work environments were associated with lower occupational stress and better somatic symptoms. However, WPV was more likely to influence occupational stress and health in healthy work environments. Based on the findings, reducing effort and overcommitment, increasing rewards, and improving the nursing work environment can alleviate health problems caused by work stressors among nurses.

**Nomenclature**
WPV: Workplace violence
ED: Emergency department
ERI: Effort–reward imbalance
SEM: Structural equation modeling
RMSEA: Root mean square error of approximation
TLI: Tucker–Lewis index
CFI: Comparative fit index
SRMR: Standardized root mean square residual
AIC: Akaike information criterion
SD: Standard deviation
CI: Confidence interval.

## Disclosure

The authors have nothing to report.

## Conflicts of Interest

The authors declare no conflicts of interest.

## Author Contributions

Xiaoli Chen conceived the study design, summarized the data, performed the statistical analysis, and drafted the manuscript. Hao Zhang summarized the data, performed the statistical analysis, and drafted the manuscript. Luying Zhong performed the statistical analysis and interpreted the data. Dongmei Diao and Ling Zhu collected the data. Lei Ye conceived the study design and revised the manuscript. Xiaoli Chen and Hao Zhang contributed equally to this article.

## Funding

This work was supported financially by grants from the Sichuan Science and Technology Program (No. 2023YFS0240).

## Supporting Information

Additional supporting information can be found online in the Supporting Information section.

## Supporting information


**Supporting Information 1** Appendix S1: This figure shows the research hypothesis that the path mechanism of violence affects occupational health of nursing staff through occupational stress. The hypothesis framework visually represents the evolution from the theoretical framework to the conceptual framework.

**Supporting Information 2** Appendix S2: The result of confirmatory factor analysis (CFA) validates the structural validity of workplace violence, occupational stress, work environment, and somatic symptom. The confirmatory factor analysis demonstrated good fit for the latent variables: all RMSEAs are < 0.1 and CFI > 0.9.

## Data Availability

The data that support the findings of this study are available from the corresponding author upon reasonable request.
