# Supplementary material for: Effects of Workplace Violence on Emergency Nurses’ Health: A Mediating and Moderating Role of Occupational Stress and the Work Environment
Source: J Nurs Manag. 2025 Dec 18;2025:8813003. doi: 10.1155/jonm/8813003 (PMC12714085; doi:10.1155/jonm/8813003)
Supplement: Supplementary file 1 — Supporting Information 1 Appendix S1: This figure shows the research hypothesis that the path mechanism of violence affects occupational health of nursing staff through occupational stress. The hypothesis framework visually represents the evolution from the theoretical framework to the conceptual framework. [file JONM-2025-8813003-s001.pptx]

## Slide 1
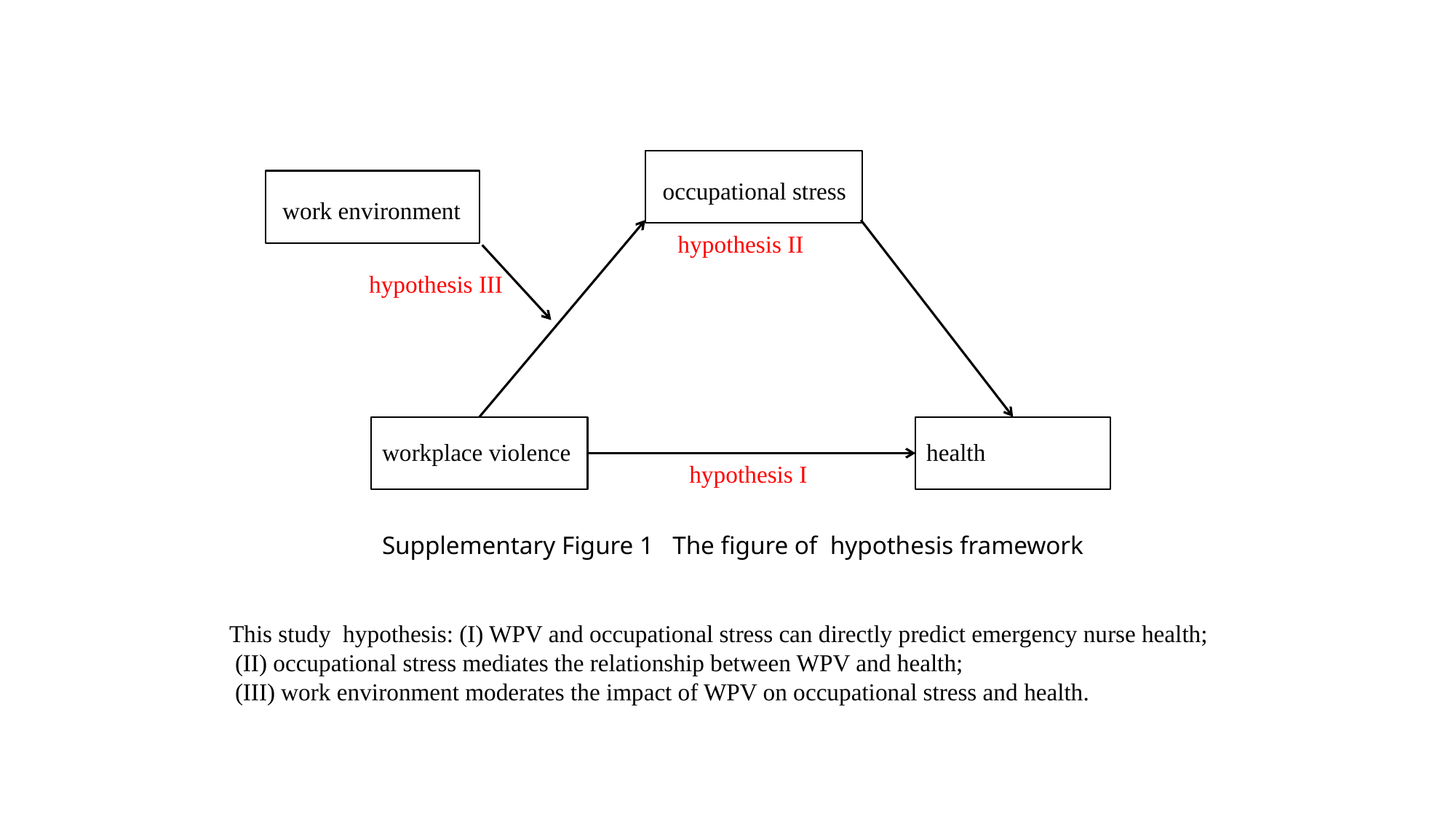

occupational stress
 work environment
hypothesis II
hypothesis III
workplace violence
health
hypothesis I
Supplementary Figure 1 The figure of hypothesis framework
This study hypothesis: (I) WPV and occupational stress can directly predict emergency nurse health;
 (II) occupational stress mediates the relationship between WPV and health;
 (III) work environment moderates the impact of WPV on occupational stress and health.
