# Supplementary material for: Effects of Workplace Violence on Emergency Nurses’ Health: A Mediating and Moderating Role of Occupational Stress and the Work Environment
Source: J Nurs Manag. 2025 Dec 18;2025:8813003. doi: 10.1155/jonm/8813003 (PMC12714085; doi:10.1155/jonm/8813003)
Supplement: Supplementary file 2 — Supporting Information 2 Appendix S2: The result of confirmatory factor analysis (CFA) validates the structural validity of workplace violence, occupational stress, work environment, and somatic symptom. The confirmatory factor analysis demonstrated good fit for the latent variables: all RMSEAs are < 0.1 and CFI > 0.9. [file JONM-2025-8813003-s002.docx]

| **The result of Confirmatory Factor Analysis** | | | | | |
| --- | --- | --- | --- | --- | --- |
| Scale | $\chi^{2}$/*df* | RMSEA | CFI | TLI | SRMR |
| Workplace violence | 0.000/0 | 0.000 | 1.000 | 1.000 | 0.000 |
| Occupational stress | 1847.360/205 | 0.072 | 0.911 | 0.900 | 0.067 |
| Nursing work environment | 1131.794/278 | 0.045 | 0.963 | 0.957 | 0.037 |
| Somatic Symptom | 1647.641/169 | 0.075 | 0.911 | 0.900 | 0.043 |


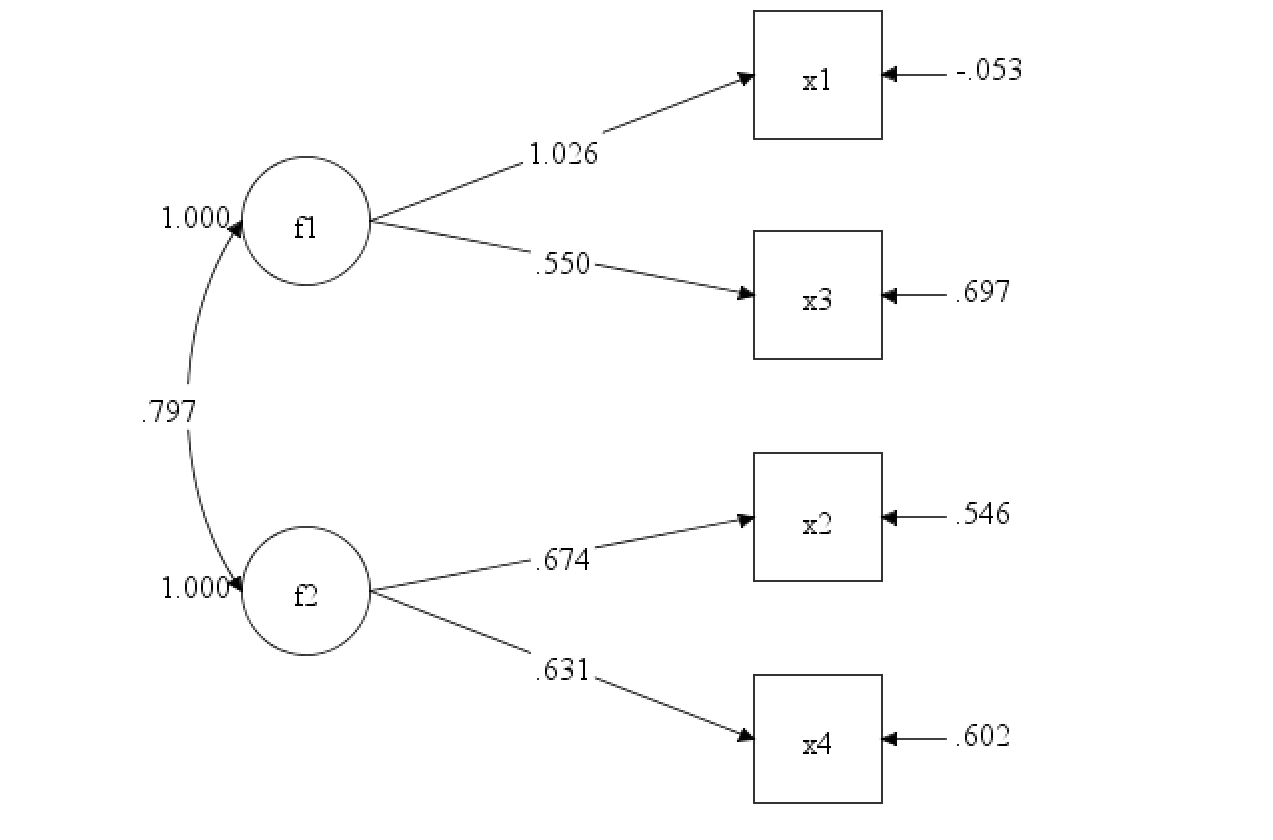


**Table 1 Workplace violence of Confirmatory Factor Analysis**

**
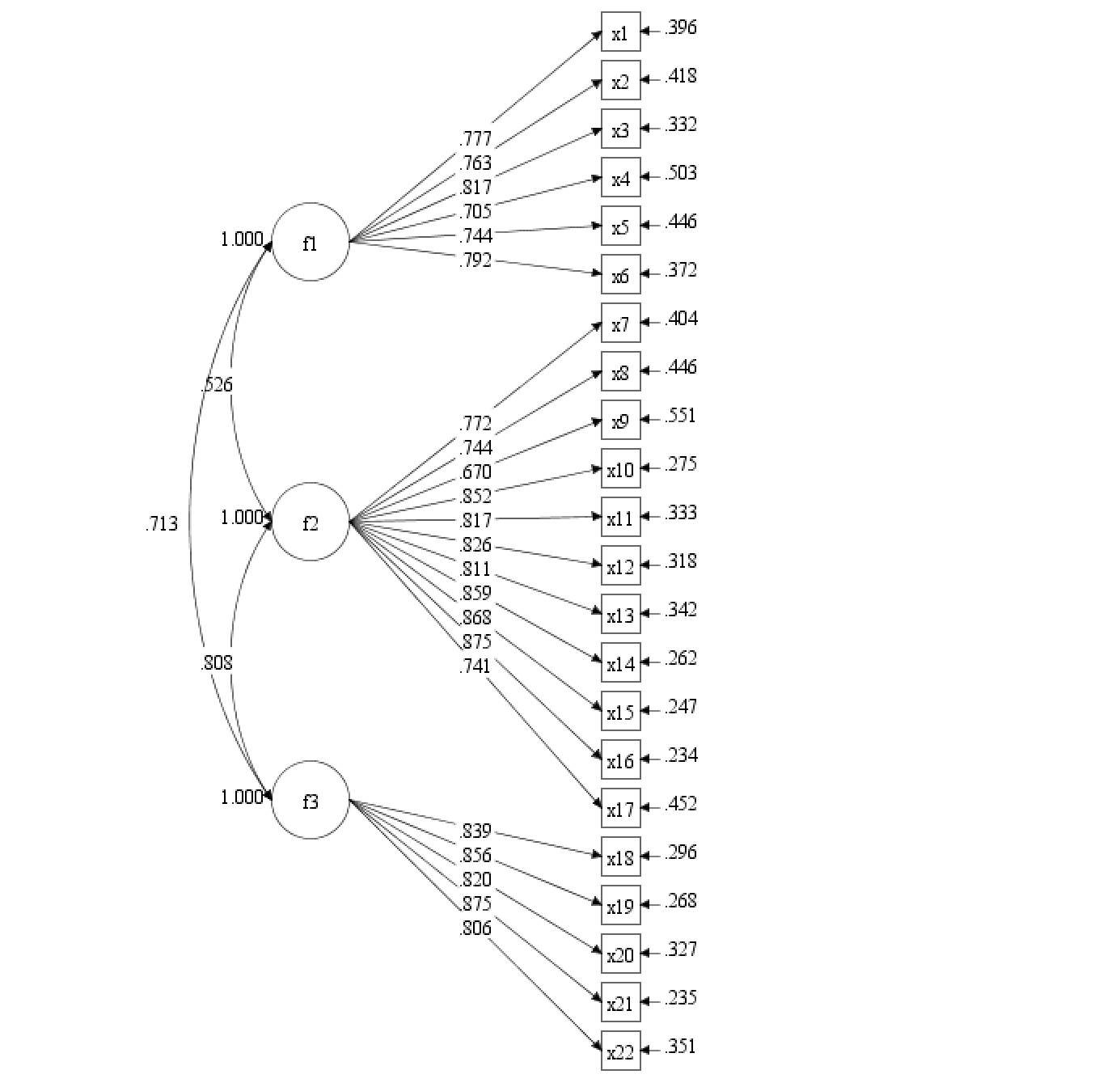
**

**Table 2 Occupational stress of Confirmatory Factor Analysis**

**
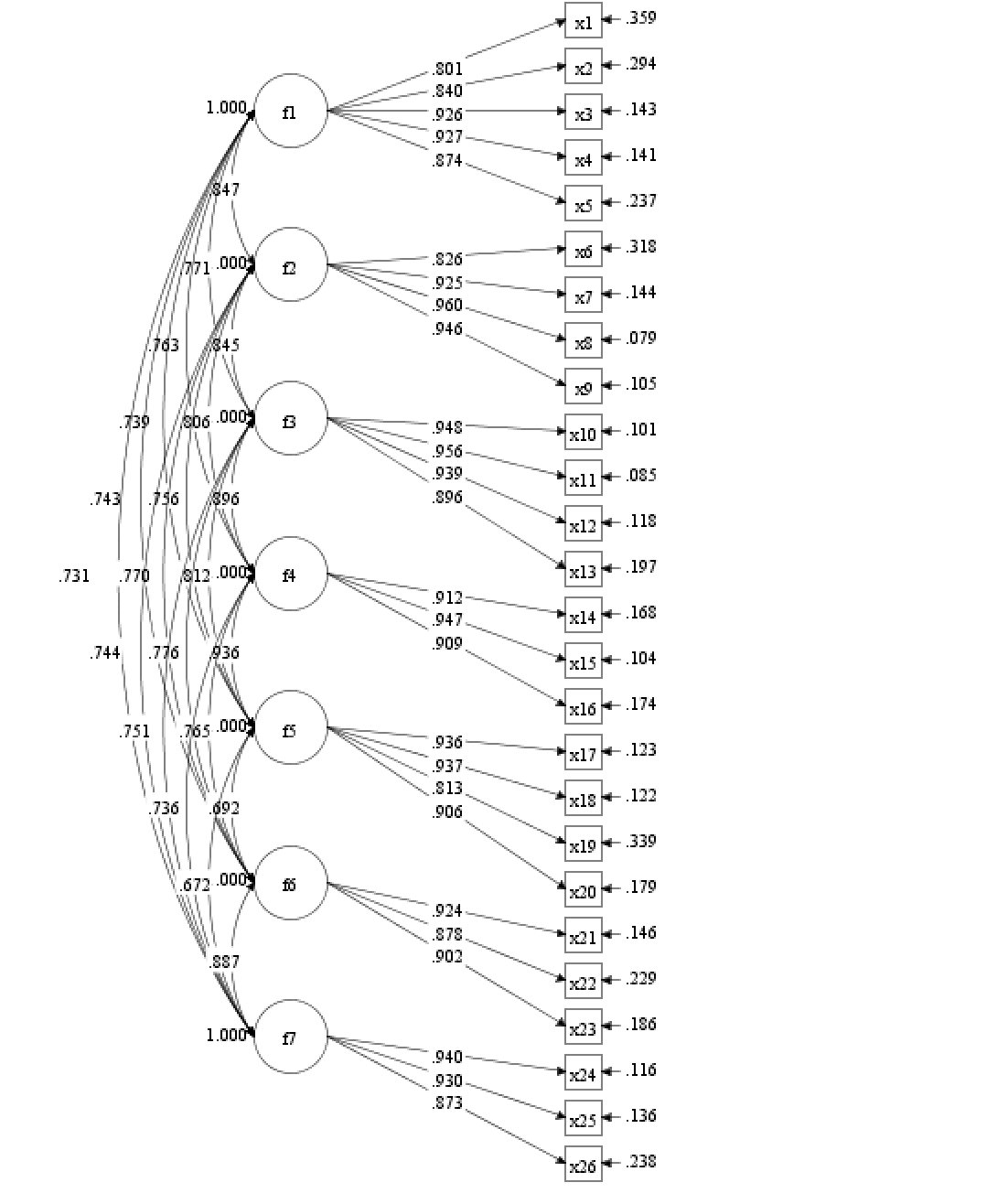
**

**Table 3 Nursing work environment of Confirmatory Factor Analysis**

**
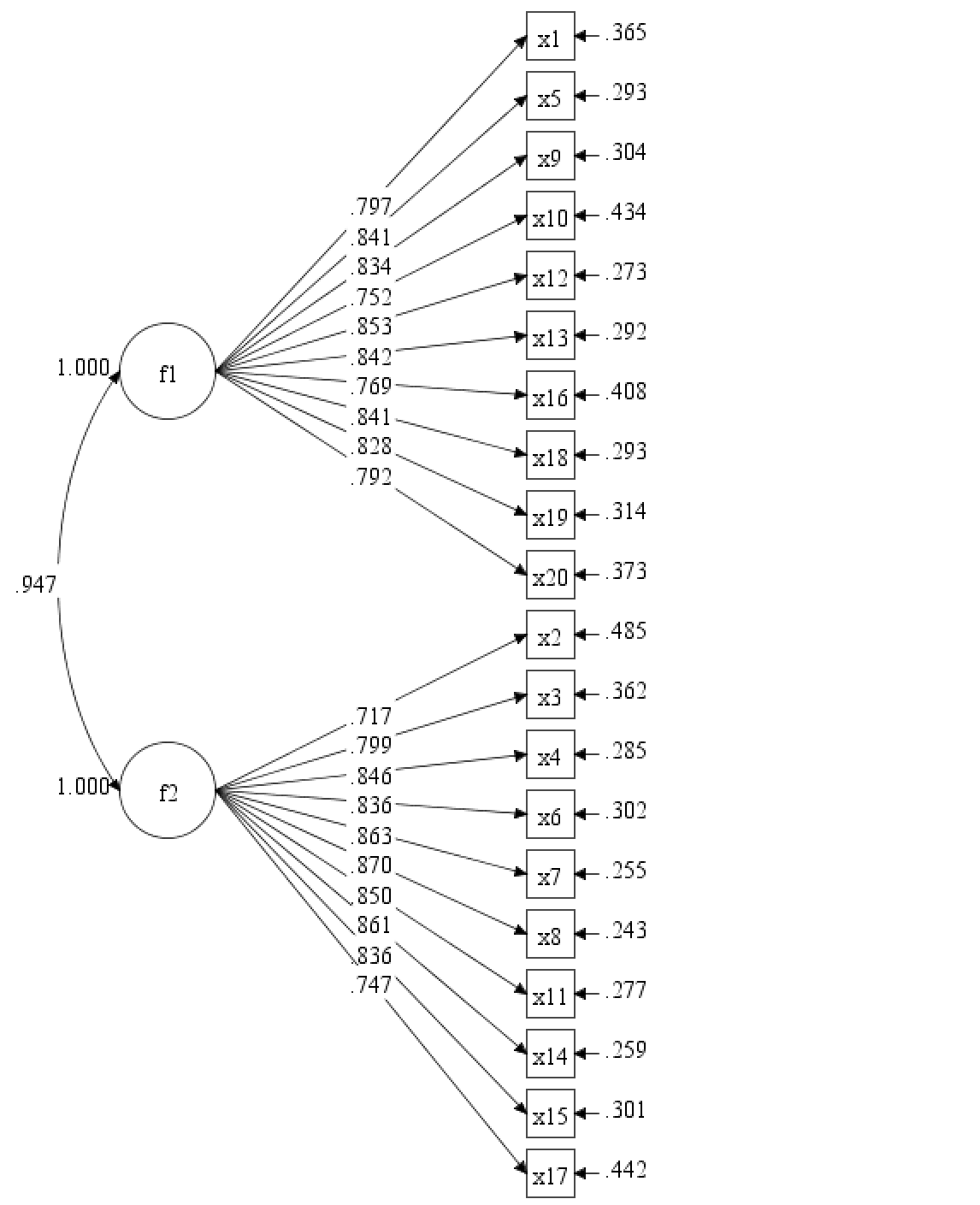
**

**Table 4 Somatic Symptom of Confirmatory Factor Analysis**
